# Supplementary material for: Comparative Outcomes of Living and Deceased Donor Liver Transplantation in Adults: A Systematic Review and Meta-Analysis
Source: J Clin Med. 2025 Dec 28;15(1):241. doi: 10.3390/jcm15010241 (PMC12786740; doi:10.3390/jcm15010241)
Supplement: Supplementary file 1 [file jcm-15-00241-s001.zip › Supplementary Material Table S1 [SOF table].pdf]

Summary of findings:

Patient or population: adult population (≥18 years)

Setting:

Intervention: LDLT

Comparison: DDLT

Outcomes	Anticipated absolute effects* (95% CI)		Relative effect (95% CI)	№ of participants (studies)	Certainty of the evidence (GRADE)	Comments
	Risk with DDLT	Risk with LDLT				
1 year patient survival	844 per 1,000	**898 per 1,000** (929 to 854)	**OR 1.62** (1.08 to 2.43)	17242 (11 non-randomised studies)	⊕◯◯◯ Very low[a,b,c,d,e,f]	898 per 1,000 with LDLT vs 844 per 1,000 with DDLT.This corresponds to an OR of 1.62 (95% CI 1.08–2.43).The difference is statistically significant.→ 54 more people per 1,000 survived at 1 year with LDLT compared to DDLT.
3 year patient survival	716 per 1,000	**794 per 1,000** (860 to 708)	**OR 1.53** (0.96 to 2.44)	15503 (7 non-randomised studies)	⊕◯◯◯ Very low[a,g,h,i,j,k]	794 per 1,000 with LDLT vs 716 per 1,000 with DDLT.This corresponds to an OR of 1.53 (95% CI 0.96–2.44).The difference is not statistically significant.→ No important difference in 3-year survival between LDLT and DDLT.
5 year patient survival	672 per 1,000	**761 per 1,000** (811 to 703)	**OR 1.56** (1.16 to 2.10)	17908 (10 non-randomised studies)	⊕◯◯◯ Very low[a,l,m,n,o,p]	761 per 1,000 with LDLT vs 672 per 1,000 with DDLT.This corresponds to an OR of 1.56 (95% CI 1.16–2.10).The difference is statistically significant.→ 89 more people per 1,000 survived at 5 years with LDLT compared to DDLT.
1 year graft survival	900 per 1,000	**896 per 1,000** (908 to 884)	**OR 0.96** (0.85 to 1.10)	9470 (5 non-randomised studies)	⊕⊕◯◯ Low[a,b,q,r,s,t,u]	896 per 1,000 with LDLT vs 900 per 1,000 with DDLT.This corresponds to an OR of 0.96 (95% CI 0.85–1.10).The difference is not statistically significant.→ No important difference in 1-year graft survival between LDLT and DDLT.
5 year graft survival	798 per 1,000	**825 per 1,000** (830 to 818)	**OR 1.19** (1.14 to 1.24)	9724 (6 non-randomised studies)	⊕⊕◯◯ Low[a,r,v,w,x,y]	825 per 1,000 with LDLT vs 798 per 1,000 with DDLT.This corresponds to an OR of 1.19 (95% CI 1.14–1.24).The difference is statistically significant.→ 27 more people per 1,000 had graft survival at 5 years with LDLT compared to DDLT.
Biliary Stricture	189 per 1,000	**190 per 1,000** (373 to 85)	**OR 1.01** (0.40 to 2.56)	3936 (5 non-randomised studies)	⊕◯◯◯ Very low[a,aa,ab,ac,ad,z]	190 per 1,000 with LDLT vs 189 per 1,000 with DDLT.This corresponds to an OR of 1.01 (95% CI 0.40–2.56).The difference is not statistically significant.→ No important difference in biliary stricture between LDLT and DDLT.

Summary of findings:

Patient or population: adult population (≥18 years)

Setting:

Intervention: LDLT

Comparison: DDLT

Outcomes	Anticipated absolute effects* (95% CI)		Relative effect (95% CI)	№ of participants (studies)	Certainty of the evidence (GRADE)	Comments
	**Risk with DDLT**	**Risk with LDLT**				
Biliary leakage	91 per 1,000	**384 per 1,000** (849 to 65)	**OR 6.19** (0.69 to 55.91)	3842 (4 non-randomised studies)	⊕◯◯◯ Very low[a,ae,af,ag,ah,ai,aj]	384 per 1,000 with LDLT vs 91 per 1,000 with DDLT.This corresponds to an OR of 6.19 (95% CI 0.69–55.91).The difference is not statistically significant.→ Although point estimate suggests more leakage with LDLT, the result is highly uncertain due to wide CI.
Hepatic artery thrombosis	27 per 1,000	**42 per 1,000** (65 to 26)	**OR 1.55** (0.97 to 2.47)	2089 (4 non-randomised studies)	⊕◯◯◯ Very low[a,ak,al,am,an,r]	42 per 1,000 with LDLT vs 27 per 1,000 with DDLT.This corresponds to an OR of 1.55 (95% CI 0.97–2.47).The difference is not statistically significant.→ No important difference in hepatic artery thrombosis between LDLT and DDLT.
Retransplantations	39 per 1,000	**56 per 1,000** (176 to 16)	**OR 1.46** (0.40 to 5.28)	4467 (5 non-randomised studies)	⊕◯◯◯ Very low[a,ao,ap,aq,ar,as]	56 per 1,000 with LDLT vs 39 per 1,000 with DDLT.This corresponds to an OR of 1.46 (95% CI 0.40–5.28).The difference is not statistically significant.→ No important difference in re-transplantation rates between LDLT and DDLT.
Infections	333 per 1,000	**209 per 1,000** (531 to 61)	**OR 0.53** (0.13 to 2.27)	8121 (4 non-randomised studies)	⊕◯◯◯ Very low[a,at,au,av,aw,ax]	209 per 1,000 with LDLT vs 333 per 1,000 with DDLT.This corresponds to an OR of 0.53 (95% CI 0.13–2.27).The difference is not statistically significant.→ No important difference in infection rates between LDLT and DDLT.

***The risk in the intervention group** (and its 95% confidence interval) is based on the assumed risk in the comparison group and the **relative effect** of the intervention (and its 95% CI).

CI: confidence interval; **OR:** odds ratio

GRADE Working Group grades of evidence
High certainty: we are very confident that the true effect lies close to that of the estimate of the effect.
Moderate certainty: we are moderately confident in the effect estimate: the true effect is likely to be close to the estimate of the effect, but there is a possibility that it is substantially different.
Low certainty: our confidence in the effect estimate is limited: the true effect may be substantially different from the estimate of the effect.
Very low certainty: we have very little confidence in the effect estimate: the true effect is likely to be substantially different from the estimate of effect.

Explanations

a. Risk of bias was assessed using the Downs and Black checklist. The following thresholds were applied: scores of 22–32 were considered high quality and corresponded to a 'not serious' rating for risk of bias in GRADE; scores of 16–21 indicated moderate quality and were rated as 'serious'; scores below 16 were considered low quality and rated as 'very serious.' These classifications were agreed upon by the review authors, acknowledging that no universally accepted guidelines currently exist for translating Downs and Black quantitative scores into GRADEpro's qualitative risk of bias categories.

b. Five of the eleven included studies were rated as high quality, contributing around 30% of the total weight. The remaining studies were of moderate quality, but none were rated low quality. The two highest-weighted moderate studies (Hu and Ziogas) were just below the high-quality threshold and showed no major methodological concerns. Given that no studies had serious flaws and high-quality studies contributed meaningfully to the findings, the overall risk of bias was rated as not serious.

c. Substantial heterogeneity was observed (I^2 = 58%), which exceeds the commonly accepted threshold of 50% for concern. Therefore, the evidence was downgraded for very serious inconsistency

d. Indirectness was downgraded by one level. Seven of the eleven studies did not match the target population, including Ninomiya (HCC), HU (HCC), Chok (MELD ≥35), Barbers (NASH), J.M. Kim (HCV), Kwon (≥70 years), Wong (HCC), and Ziogas (PSC/PBC), contributing approximately 67% of the total weight in the analysis.

e. A downgrade by one point for imprecision was applied because the 95% confidence interval for the pooled odds ratio (1.62 [1.08, 2.43]) extends beyond the upper clinical decision threshold of 1.25, indicating potential for no clinically meaningful effect. Although the total number of events exceeds 300, the confidence interval remains wide, justifying a single-level downgrade.

f. A funnel plot and Egger's regression test were conducted to assess publication bias. The p-value was 0.221, indicating no significant asymmetry and suggesting that publication bias is unlikely.

g. For the 3-year survival outcome, five studies were rated moderate quality (Ninomiya, HU, Barbers, Humar, Ziogas) and two were high quality (Chok, Wong). The highest-weighted studies were moderate in quality but methodologically sound. As no studies were low quality or showed critical flaws, the risk of bias was judged not serious.

h. Substantial heterogeneity was observed (I^2 = 65%), which is above the commonly used threshold of 50% for concern. As a result, the certainty of evidence was downgraded by two levels for serious inconsistency.

i. Indirectness was downgraded by one level. Five of the seven studies did not match the review's population, including Ninomiya (HCC), HU (HCC), Barbers (NASH), Chok (MELD ≥35), and Wong (HCC), contributing approximately 56% of the total weight in the analysis.

j. A downgrade by one point for imprecision was applied because the 95% confidence interval for the pooled odds ratio (1.53 [0.96, 2.44]) extends beyond the upper clinical decision threshold of 1.25, indicating uncertainty regarding a meaningful benefit. Although the total number of events (11,360) is sufficient, the wide confidence interval justifies a downgrade.

k. Fewer than 10 studies were included, so formal funnel plot testing is not applicable. However, the included studies show consistent findings across various centers and sample sizes with no evidence of selective publication, so publication bias is unlikely.

l. The risk of bias is rated "not serious" because while 5 moderate-quality studies (scoring 19-21, just below the 22+ high-quality threshold) contribute 60.3% weight, their limitations were minor (e.g., reporting details) rather than critical flaws. The 5 high-quality studies (39.7% weight) include large, rigorous trials (e.g., Goto, Azoulay) that confirm the findings. The consistent effect direction across all studies, without evidence of critical bias, supports this judgment. No downgrade is warranted.

m. Heterogeneity was substantial (I^2 = 64%), exceeding the typical threshold of 50%. Therefore, the certainty of evidence was downgraded by two level for serious inconsistency

n. Indirectness was downgraded by one level. Eight of the ten studies did not match the target population, including Ninomiya (HCC), HU (HCC), Barbers (NASH), Azoulay (HCC), Chok (MELD ≥35), Kwon (≥70 years), Wong (HCC), and Braun (ALD), contributing approximately 72% of the total weight in the analysis.

o. Imprecision was downgraded by one point because the 95% confidence interval (1.56 [1.16 to 2.10]) exceeded the upper decision threshold of 1.25, introducing uncertainty about the magnitude of effect despite not crossing the line of no effect. Additionally, the total number of events (12413) met the optimal information size.

p. A funnel plot and Egger's regression test were conducted to assess publication bias. The p-value was 0.303, indicating no significant asymmetry and suggesting that publication bias is unlikely.

q. The risk of bias is rated "not serious" because 33.5% of weight comes from Goto (high-quality, score=24), while 61.7% comes from Ziogas (moderate-quality, score=19). Though Ziogas dominates the weight, its score of 19 only narrowly misses the high-quality threshold (22+), with limitations being minor reporting issues rather than critical methodological flaws. The remaining 4.8% weight comes from three small moderate-quality studies.

r. Heterogeneity was negligible (I^2 = 0%), indicating consistency across studies. Therefore, the certainty of evidence was not downgraded for inconsistency.

s. Indirectness was not downgraded. Although three of the five studies had restricted populations—Barbers (NASH), Chok (MELD ≥35), and Kwon (≥70 years)—their combined contribution to the total weight was minimal (4.8%), while the remaining two studies, Ziogas and Goto, represented broad adult populations and accounted for over 95% of the total weight.

t. Imprecision was not downgraded because the 95% confidence interval (0.96 [0.85 to 1.10]) lies entirely within the decision threshold range of 0.75 to 1.25, and the total number of events (8518) meets the optimal information size, providing sufficient precision.

u. Although fewer than 10 studies are included, the analysis involves a range of small and large studies (e.g. Ziogas and Goto hold the majority of the weight), and there is no visible evidence of asymmetry or selective publication. In the absence of funnel plot reliability, and given consistency across study sizes and sources, publication bias is unlikely.

v. The risk of bias is "not serious" because while moderate-quality studies contribute most weight (63.8%), the dominant Ziogas study (54.2%) only narrowly missed high-quality status (score=19/32) with minor limitations.

w. Indirectness was not downgraded. Although four of the six studies—Barbers (NASH), Chok (MELD ≥35), Kwon (≥70 years), and Braun (ALD)—had restricted populations, they collectively contributed only around 9.6% of the total weight, while the remaining two broad-population studies, Ziogas and Goto, accounted for over 90% of the analysis.

x. Imprecision was not downgraded because the 95% confidence interval (1.19 [1.14 to 1.24]) falls entirely within the predefined decision threshold range of 0.75 to 1.25, and the total number of events (7791) meets the optimal information size, indicating adequate precision.

y. This outcome includes six studies, below the optimal threshold for formal funnel plot assessment. However, the distribution of effect sizes is balanced, and two high-weight studies (Ziogas and Goto) dominate the estimate, reducing the risk that selective publication among smaller studies would substantially alter the findings. No clear indication of publication bias is present.

z. The risk of bias was "not serious" because 3/5 studies included were high quality and they accounted for 62.4% of the total cases.

aa. Heterogeneity was substantial (I^2 = 82%), indicating considerable variability in effect estimates across studies. Therefore, the certainty of evidence was downgraded by two levels for serious inconsistency.

ab. Indirectness was not downgraded. Although Barbers (NASH) and Chok (MELD ≥35) involved restricted populations, their combined contribution to the total weight was low (approximately 17%), while the remaining three studies—Samstein, Humar, and Amara—included general adult populations and accounted for over 80% of the weight.

ac. Imprecision was downgraded by one point because the 95% confidence interval (1.01 [0.40 to 2.56]) crosses both decision thresholds of 0.75 and 1.25, indicating uncertainty about the magnitude and direction of the effect. However, no further downgrade was made since the total number of events (773) exceeds optimal information size.

ad. Although only five studies are included, which limits formal assessment, the distribution of effect sizes is balanced and driven primarily by two large studies (Samstein and Amara), minimizing the likelihood that selective publication bias among smaller studies would distort the overall result.

ae. Risk of bias was "not serious" although half of the studies were of moderate quality, 53.5% of the cases were from the high quality studies. Also, the moderate quality studies were close to achieving high quality and had no major flaws warranting a downgrade.

af. "Heterogeneity was substantial (I^2 = 96%), reflecting extreme variability among the included studies. This high degree of inconsistency warrants downgrading the certainty of evidence by two levels.

ag. Indirectness was not downgraded. Although Barbers (NASH) represented a restricted population, it contributed only 19.9% of the total weight. The remaining three studies—Samstein, Humar, and Amara—included broad adult populations and together contributed over 80% of the total weight.

ah. Imprecision was downgraded by two points because the 95% confidence interval (6.19 [0.69 to 55.91]) is extremely wide and crosses both decision thresholds of 0.75 and 1.25, indicating substantial uncertainty in the effect estimate. Additionally, the total number of events (270 + 263 = 533) is just above the optimal information size, but the imprecision remains very serious due to the extreme range of the CI.

ai. Downgrade by 1 point. Although fewer than 10 studies are included (limiting formal assessment), all studies show effects favoring LDLT with no neutral or opposite findings, suggesting asymmetry. This skewed distribution, combined with small-study effects and high heterogeneity, raises concern for possible publication bias.

aj. Although the point estimate (OR = 6.19) suggests a very large effect, the wide 95% CI [0.69, 55.91] crosses the line of no effect and indicates substantial imprecision. Therefore, the criteria for upgrading based on large or very large effect are not met.

ak. Risk of bias is "not serious" because although most of the studies were moderate, the majority of the cases (67.4%) came from the high quality study (Samstein et al.)

al. Indirectness was not downgraded. Only one study, Barbers (NASH), did not align with the target population and contributed a small proportion of the total weight (2.9%), which does not justify a downgrade.

am. Imprecision was downgraded by two points because the confidence interval (0.97 to 2.47) crosses the upper decision threshold of 1.25, indicating uncertainty about whether the true effect is clinically meaningful. Additionally, the total number of events (77) is far below the optimal information size of 300, further supporting serious concerns about imprecision.

an. No downgrade. Fewer than 10 studies are included, limiting funnel plot assessment, but the distribution of results is reasonably symmetric with variation on both sides of the line of no effect. The weights are also fairly concentrated in the larger studies, reducing concern for small-study publication bias.

ao. Risk of bias was "not serious" because although 3/5 studies included were of moderate quality, they were close to high quality in their score and didn't have any major flaws. Additionally high quality studies accounted for 47.6% of the cases further strengthening the conclusion.

ap. Heterogeneity was substantial ($I^2 = 79\%$), indicating considerable variability between study results. As this level of heterogeneity may impact the reliability of the pooled estimate, the certainty of evidence was downgraded by two levels for very serious inconsistency.

aq. Indirectness was not downgraded. Only one study, Barbers (NASH), did not match the general adult liver transplant population. It contributed a small proportion of the overall weight (6.7%), which does not significantly influence the pooled estimate.

ar. Imprecision was downgraded by two points because the confidence interval (1.46 [0.40 to 5.28]) is very wide and crosses both the lower and upper decision thresholds (0.75 and 1.25), indicating serious uncertainty about the effect estimate. Additionally, the total number of events (192) is below the optimal information size threshold of 300, further supporting very serious imprecision.

as. Downgrade by one point. Fewer than 10 studies are included, and there is visible asymmetry in the forest plot, with effect sizes skewed to the right suggesting potential small-study effects or selective publication.

at. Risk of bias is "not serious" although most of the studies were of moderate quality, they were close to high quality in terms of scoring on DBC and they had no major flaws warranting a downgrade.

au. Heterogeneity was substantial ($I^2 = 90\%$), indicating considerable inconsistency in effect estimates across studies. This justifies downgrading the certainty of evidence by two levels for serious inconsistency.

av. Indirectness was downgraded by one level as HU (HCC-only) did not match the target population and contributed 32% of the total weight; the remaining studies matched the PICO and held the majority of the weight.

aw. Imprecision was downgraded by one point because the confidence interval (0.53 [0.13 to 2.27]) crosses both the lower and upper decision thresholds (0.75 and 1.25), indicating uncertainty about the magnitude of the effect. However, the total number of events (2693) exceeds the optimal information size, preventing a second level of downgrade.

ax. Downgrade by one point. Fewer than 10 studies are included and the distribution is asymmetrical, with three studies clustered on the left of the no-effect line and only one small study (Lapisatepun) on the right, indicating possible publication bias.
